# Supplementary material for: Level of anxiety and depression among healthcare workers in Saudi Arabia during the COVID-19 pandemic
Source: PeerJ. 2022 Dec 1;10:e14246. doi: 10.7717/peerj.14246 (PMC9744137; doi:10.7717/peerj.14246)
Supplement: Supplemental Information 2 [file peerj-10-14246-s002.docx]

**Codebook**

| **gender** | | | | |
| --- | --- | --- | --- | --- |
|  | | Value | Count | Percent |
| Standard Attributes | Position | 2 |  |  |
|  | Label | What is your gender? |  |  |
|  | Type | Numeric |  |  |
|  | Format | F40 |  |  |
|  | Measurement | Nominal |  |  |
|  | Role | Input |  |  |
| Valid Values | 1 | Male | 135 | 41.4% |
|  | 2 | Female | 191 | 58.6% |

| **marital** | | | | |
| --- | --- | --- | --- | --- |
|  | | Value | Count | Percent |
| Standard Attributes | Position | 3 |  |  |
|  | Label | What is your marital status? |  |  |
|  | Type | Numeric |  |  |
|  | Format | F40 |  |  |
|  | Measurement | Nominal |  |  |
|  | Role | Input |  |  |
| Valid Values | 1 | Single | 141 | 43.3% |
|  | 2 | Engaged | 5 | 1.5% |
|  | 3 | Married | 161 | 49.4% |
|  | 4 | Divorced or Widowed | 19 | 5.8% |

| **ses** | | | | |
| --- | --- | --- | --- | --- |
|  | | Value | Count | Percent |
| Standard Attributes | Position | 4 |  |  |
|  | Label | In general, how would you describe your economic status? |  |  |
|  | Type | Numeric |  |  |
|  | Format | F40 |  |  |
|  | Measurement | Ordinal |  |  |
|  | Role | Input |  |  |
| Valid Values | 1 | Poor | 7 | 2.1% |
|  | 2 | Good | 143 | 43.9% |
|  | 3 | Very good | 137 | 42.0% |
|  | 4 | Excellent | 39 | 12.0% |

| **national** | | | | |
| --- | --- | --- | --- | --- |
|  | | Value | Count | Percent |
| Standard Attributes | Position | 5 |  |  |
|  | Label | What is your nationality? |  |  |
|  | Type | Numeric |  |  |
|  | Format | F40 |  |  |
|  | Measurement | Nominal |  |  |
|  | Role | Input |  |  |
| Valid Values | 1 | Saudi | 309 | 94.8% |
|  | 2 | non-Saudi | 17 | 5.2% |

| **facility** | | | | |
| --- | --- | --- | --- | --- |
|  | | Value | Count | Percent |
| Standard Attributes | Position | 6 |  |  |
|  | Label | What type of healthcare facility do you work in? |  |  |
|  | Type | Numeric |  |  |
|  | Format | F40 |  |  |
|  | Measurement | Nominal |  |  |
|  | Role | Input |  |  |
| Valid Values | 1 | Public/governmental | 293 | 89.9% |
|  | 2 | Private | 33 | 10.1% |

| **profession** | | | | |
| --- | --- | --- | --- | --- |
|  | | Value | Count | Percent |
| Standard Attributes | Position | 7 |  |  |
|  | Label | What is your profession? |  |  |
|  | Type | Numeric |  |  |
|  | Format | F40 |  |  |
|  | Measurement | Nominal |  |  |
|  | Role | Input |  |  |
| Valid Values | 1 | Physician | 113 | 34.7% |
|  | 2 | Nurse | 71 | 21.8% |
|  | 3 | Pharmacist | 33 | 10.1% |
|  | 4 | Working in labs | 33 | 10.1% |
|  | 5 | Other | 76 | 23.3% |

| **type_hos** | | | | |
| --- | --- | --- | --- | --- |
|  | | Value | Count | Percent |
| Standard Attributes | Position | 8 |  |  |
|  | Label | Where do you work? |  |  |
|  | Type | Numeric |  |  |
|  | Format | F40 |  |  |
|  | Measurement | Nominal |  |  |
|  | Role | Input |  |  |
| Valid Values | 1 | A primary healthcare center | 68 | 20.9% |
|  | 2 | Secondary or tertiary hospital | 132 | 40.5% |
|  | 3 | Specialized hospital | 87 | 26.7% |
|  | 4 | Polyclinic | 4 | 1.2% |
|  | 5 | Lab | 13 | 4.0% |
|  | 6 | Other | 22 | 6.7% |

| **area** | | | | |
| --- | --- | --- | --- | --- |
|  | | Value | Count | Percent |
| Standard Attributes | Position | 9 |  |  |
|  | Label | In which area do you mostly work? |  |  |
|  | Type | Numeric |  |  |
|  | Format | F40 |  |  |
|  | Measurement | Nominal |  |  |
|  | Role | Input |  |  |
| Valid Values | 1 | ER | 46 | 14.1% |
|  | 2 | Ward | 79 | 24.2% |
|  | 3 | ICU | 25 | 7.7% |
|  | 4 | Labs | 34 | 10.4% |
|  | 5 | Other | 142 | 43.6% |

| **covid_1** | | | | |
| --- | --- | --- | --- | --- |
|  | | Value | Count | Percent |
| Standard Attributes | Position | 11 |  |  |
|  | Label | What is your involvement with COVID-19? - Selected Choice |  |  |
|  | Type | Numeric |  |  |
|  | Format | F40 |  |  |
|  | Measurement | Ordinal |  |  |
|  | Role | Input |  |  |
| Valid Values | 1 | I have not been involved in any COVID-19 case. | 151 | 46.3% |
|  | 2 | Diagnosis | 48 | 14.7% |
|  | 3 | Treatment | 35 | 10.7% |
|  | 4 | Nursing care | 28 | 8.6% |
|  | 5 | Other (please specify) | 64 | 19.6% |

| **covid_1_5_TEXT** | | | | |
| --- | --- | --- | --- | --- |
|  | | Value | Count | Percent |
| Standard Attributes | Position | 12 |  |  |
|  | Label | What is your involvement with COVID-19? - Other (please specify) - Text |  |  |
|  | Type | String |  |  |
|  | Format | A2000 |  |  |
|  | Measurement | Nominal |  |  |
|  | Role | Input |  |  |
| Valid Values |  |  | 269 | 82.5% |
|  | ... |  | 1 | 0.3% |
|  | Assessment of all suspected cases, admitting them, diagnosis and treatment |  | 1 | 0.3% |
|  | Both diagnosis and treatment |  | 1 | 0.3% |
|  | Collecting samples |  | 1 | 0.3% |
|  | Consultation for a non Covid19 related pathology |  | 1 | 0.3% |
|  | Contact with nurses and doctors and dispense the med |  | 1 | 0.3% |
|  | Dental emergency for suspected COVID-19 patients |  | 1 | 0.3% |
|  | Dental service |  | 1 | 0.3% |
|  | Dentist, treatment of patient who might have COVID19 |  | 1 | 0.3% |
|  | Diagnosis and treatment |  | 2 | 0.6% |
|  | Diagnosis,treatment |  | 1 | 0.3% |
|  | Doing ultrasound scan for them if its need |  | 1 | 0.3% |
|  | E.M.S |  | 1 | 0.3% |
|  | Epidemiologists |  | 1 | 0.3% |
|  | Follow up all Exposed HCW educate them |  | 1 | 0.3% |
|  | food service |  | 1 | 0.3% |
|  | handling with sample for patient who diagnosis or suspect for covid-19 |  | 1 | 0.3% |
|  | I performed a procedure to a patient which was later diagnosed with COVID 19 |  | 1 | 0.3% |
|  | I was suspicted to have came out negative |  | 1 | 0.3% |
|  | I work in eye clinic (the eye has been considered as one of possible coronavirus transmitting media) |  | 1 | 0.3% |
|  | I’m a dietitian, so planning tube feeding to meet patients nutritional requirements |  | 1 | 0.3% |
|  | In command & control center of COVID 19 |  | 1 | 0.3% |
|  | Infection control |  | 1 | 0.3% |
|  | My responsibility is to identifying the suspected cases and transferring them for swabbing |  | 1 | 0.3% |
|  | None |  | 1 | 0.3% |
|  | Nutrition - tube feeding |  | 1 | 0.3% |
|  | OR |  | 1 | 0.3% |
|  | Our patients diagnose with it |  | 1 | 0.3% |
|  | Participating in covid 19 swaping |  | 1 | 0.3% |
|  | Patient experience |  | 1 | 0.3% |
|  | Provide and monitor POCT services in all hospital wards |  | 1 | 0.3% |
|  | Provide anesthesia for covid 19 positive patient |  | 1 | 0.3% |
|  | Providing anesthesia (including air way management and ventilation to pregnant woman suspected or confirmed case of COVID 19 |  | 1 | 0.3% |
|  | Quarantine |  | 1 | 0.3% |
|  | Quarantine physician + ER + family medicine clinic |  | 1 | 0.3% |
|  | Radiological diagnosis |  | 1 | 0.3% |
|  | Radiology, medical imaging |  | 1 | 0.3% |
|  | Receiving COVID-19 samples and shipping them to the reference lab |  | 1 | 0.3% |
|  | Respiratory Care |  | 1 | 0.3% |
|  | Respiratory Therapist |  | 1 | 0.3% |
|  | Screening |  | 1 | 0.3% |
|  | Sort and take vital signs |  | 1 | 0.3% |
|  | Sorting of contacts |  | 1 | 0.3% |
|  | spacemens patient (sample) |  | 1 | 0.3% |
|  | Supervisor |  | 1 | 0.3% |
|  | Suspected cases |  | 1 | 0.3% |
|  | Swabs taking for suspected/ or symptomatic patients |  | 1 | 0.3% |
|  | Transfers or front disk triage |  | 1 | 0.3% |
|  | Translation |  | 1 | 0.3% |
|  | Treating team of suspected case |  | 1 | 0.3% |
|  | Triage respiratory and observation |  | 1 | 0.3% |
|  | Triaging |  | 1 | 0.3% |
|  | triaging, suspicion, diagnosis & treatment with also transportation to other healthcare facility |  | 1 | 0.3% |
|  | Work in near the area of isolation |  | 1 | 0.3% |
|  | X-ray |  | 2 | 0.6% |

| **covid_3** | | | | |
| --- | --- | --- | --- | --- |
|  | | Value | Count | Percent |
| Standard Attributes | Position | 14 |  |  |
|  | Label | What is the highest level of severity of COVID-19 cases you dealt with? |  |  |
|  | Type | Numeric |  |  |
|  | Format | F40 |  |  |
|  | Measurement | Ordinal |  |  |
|  | Role | Input |  |  |
| Valid Values | 1 | Mild | 93 | 28.5% |
|  | 2 | Moderate | 47 | 14.4% |
|  | 3 | Severe | 24 | 7.4% |
|  | 4 | Fatal cases | 11 | 3.4% |
| Missing Values | System |  | 151 | 46.3% |

| **Anxiety_1** | | | | |
| --- | --- | --- | --- | --- |
|  | | Value | Count | Percent |
| Standard Attributes | Position | 15 |  |  |
|  | Label | Feeling nervous, anxious, or on edge |  |  |
|  | Type | Numeric |  |  |
|  | Format | F40 |  |  |
|  | Measurement | Ordinal |  |  |
|  | Role | Input |  |  |
| Valid Values | 0 | Not at all | 61 | 18.7% |
|  | 1 | Several days | 165 | 50.6% |
|  | 2 | Over half of the days | 46 | 14.1% |
|  | 3 | Nearly everyday | 54 | 16.6% |

| **Anxiety_2** | | | | |
| --- | --- | --- | --- | --- |
|  | | Value | Count | Percent |
| Standard Attributes | Position | 16 |  |  |
|  | Label | Not being able to stop or control worrying |  |  |
|  | Type | Numeric |  |  |
|  | Format | F40 |  |  |
|  | Measurement | Ordinal |  |  |
|  | Role | Input |  |  |
| Valid Values | 0 | Not at all | 102 | 31.3% |
|  | 1 | Several days | 154 | 47.2% |
|  | 2 | Over half of the days | 33 | 10.1% |
|  | 3 | Nearly everyday | 37 | 11.3% |

| **Anxiety_3** | | | | |
| --- | --- | --- | --- | --- |
|  | | Value | Count | Percent |
| Standard Attributes | Position | 17 |  |  |
|  | Label | Worrying too much about different things |  |  |
|  | Type | Numeric |  |  |
|  | Format | F40 |  |  |
|  | Measurement | Ordinal |  |  |
|  | Role | Input |  |  |
| Valid Values | 0 | Not at all | 58 | 17.8% |
|  | 1 | Several days | 158 | 48.5% |
|  | 2 | Over half of the days | 48 | 14.7% |
|  | 3 | Nearly everyday | 62 | 19.0% |

| **Anxiety_4** | | | | |
| --- | --- | --- | --- | --- |
|  | | Value | Count | Percent |
| Standard Attributes | Position | 18 |  |  |
|  | Label | Trouble relaxing |  |  |
|  | Type | Numeric |  |  |
|  | Format | F40 |  |  |
|  | Measurement | Ordinal |  |  |
|  | Role | Input |  |  |
| Valid Values | 0 | Not at all | 86 | 26.4% |
|  | 1 | Several days | 156 | 47.9% |
|  | 2 | Over half of the days | 49 | 15.0% |
|  | 3 | Nearly everyday | 35 | 10.7% |

| **Anxiety_5** | | | | |
| --- | --- | --- | --- | --- |
|  | | Value | Count | Percent |
| Standard Attributes | Position | 19 |  |  |
|  | Label | Being so restless that it is hard to sit still |  |  |
|  | Type | Numeric |  |  |
|  | Format | F40 |  |  |
|  | Measurement | Ordinal |  |  |
|  | Role | Input |  |  |
| Valid Values | 0 | Not at all | 151 | 46.3% |
|  | 1 | Several days | 120 | 36.8% |
|  | 2 | Over half of the days | 38 | 11.7% |
|  | 3 | Nearly everyday | 17 | 5.2% |

| **Anxiety_6** | | | | |
| --- | --- | --- | --- | --- |
|  | | Value | Count | Percent |
| Standard Attributes | Position | 20 |  |  |
|  | Label | Becoming easily annoyed or irritable |  |  |
|  | Type | Numeric |  |  |
|  | Format | F40 |  |  |
|  | Measurement | Ordinal |  |  |
|  | Role | Input |  |  |
| Valid Values | 0 | Not at all | 83 | 25.5% |
|  | 1 | Several days | 152 | 46.6% |
|  | 2 | Over half of the days | 56 | 17.2% |
|  | 3 | Nearly everyday | 35 | 10.7% |

| **Anxiety_7** | | | | |
| --- | --- | --- | --- | --- |
|  | | Value | Count | Percent |
| Standard Attributes | Position | 21 |  |  |
|  | Label | Feeling afraid as if something awful might happen |  |  |
|  | Type | Numeric |  |  |
|  | Format | F40 |  |  |
|  | Measurement | Ordinal |  |  |
|  | Role | Input |  |  |
| Valid Values | 0 | Not at all | 103 | 31.6% |
|  | 1 | Several days | 136 | 41.7% |
|  | 2 | Over half of the days | 46 | 14.1% |
|  | 3 | Nearly everyday | 41 | 12.6% |

| **Dprsn_1** | | | | |
| --- | --- | --- | --- | --- |
|  | | Value | Count | Percent |
| Standard Attributes | Position | 22 |  |  |
|  | Label | I feel down hearted and blue |  |  |
|  | Type | Numeric |  |  |
|  | Format | F40 |  |  |
|  | Measurement | Ordinal |  |  |
|  | Role | Input |  |  |
| Valid Values | 1 | A little of the time | 179 | 54.9% |
|  | 2 | Some of the time | 84 | 25.8% |
|  | 3 | Good part of the time | 43 | 13.2% |
|  | 4 | Most of the time | 20 | 6.1% |

| **Dprsn_2** | | | | |
| --- | --- | --- | --- | --- |
|  | | Value | Count | Percent |
| Standard Attributes | Position | 23 |  |  |
|  | Label | Morning is when I feel the best |  |  |
|  | Type | Numeric |  |  |
|  | Format | F40 |  |  |
|  | Measurement | Ordinal |  |  |
|  | Role | Input |  |  |
| Valid Values | 1 | Most of the time | 63 | 19.3% |
|  | 2 | Good part of the time | 71 | 21.8% |
|  | 3 | Some of the time | 100 | 30.7% |
|  | 4 | A little of the time | 92 | 28.2% |

| **Dprsn_3** | | | | |
| --- | --- | --- | --- | --- |
|  | | Value | Count | Percent |
| Standard Attributes | Position | 24 |  |  |
|  | Label | I have crying spells or feel like it |  |  |
|  | Type | Numeric |  |  |
|  | Format | F40 |  |  |
|  | Measurement | Ordinal |  |  |
|  | Role | Input |  |  |
| Valid Values | 1 | A little of the time | 205 | 62.9% |
|  | 2 | Some of the time | 88 | 27.0% |
|  | 3 | Good part of the time | 21 | 6.4% |
|  | 4 | Most of the time | 12 | 3.7% |

| **Dprsn_4** | | | | |
| --- | --- | --- | --- | --- |
|  | | Value | Count | Percent |
| Standard Attributes | Position | 25 |  |  |
|  | Label | I have trouble sleeping at night |  |  |
|  | Type | Numeric |  |  |
|  | Format | F40 |  |  |
|  | Measurement | Ordinal |  |  |
|  | Role | Input |  |  |
| Valid Values | 1 | A little of the time | 156 | 47.9% |
|  | 2 | Some of the time | 89 | 27.3% |
|  | 3 | Good part of the time | 29 | 8.9% |
|  | 4 | Most of the time | 52 | 16.0% |

| **Dprsn_5** | | | | |
| --- | --- | --- | --- | --- |
|  | | Value | Count | Percent |
| Standard Attributes | Position | 26 |  |  |
|  | Label | I eat as much as I used to |  |  |
|  | Type | Numeric |  |  |
|  | Format | F40 |  |  |
|  | Measurement | Ordinal |  |  |
|  | Role | Input |  |  |
| Valid Values | 1 | Most of the time | 79 | 24.2% |
|  | 2 | Good part of the time | 64 | 19.6% |
|  | 3 | Some of the time | 87 | 26.7% |
|  | 4 | A little of the time | 96 | 29.4% |

| **Dprsn_6** | | | | |
| --- | --- | --- | --- | --- |
|  | | Value | Count | Percent |
| Standard Attributes | Position | 27 |  |  |
|  | Label | I still enjoy sex |  |  |
|  | Type | Numeric |  |  |
|  | Format | F40 |  |  |
|  | Measurement | Ordinal |  |  |
|  | Role | Input |  |  |
| Valid Values | 0 | Not applicable | 158 | 48.5% |
|  | 1 | Most of the time | 43 | 13.2% |
|  | 2 | Good part of the time | 30 | 9.2% |
|  | 3 | Some of the time | 37 | 11.3% |
|  | 4 | A little of the time | 58 | 17.8% |

| **Dprsn_7** | | | | |
| --- | --- | --- | --- | --- |
|  | | Value | Count | Percent |
| Standard Attributes | Position | 28 |  |  |
|  | Label | I notice that I am losing weight |  |  |
|  | Type | Numeric |  |  |
|  | Format | F40 |  |  |
|  | Measurement | Ordinal |  |  |
|  | Role | Input |  |  |
| Valid Values | 1 | A little of the time | 236 | 72.4% |
|  | 2 | Some of the time | 47 | 14.4% |
|  | 3 | Good part of the time | 26 | 8.0% |
|  | 4 | Most of the time | 17 | 5.2% |

| **Dprsn_8** | | | | |
| --- | --- | --- | --- | --- |
|  | | Value | Count | Percent |
| Standard Attributes | Position | 29 |  |  |
|  | Label | I have trouble with constipation |  |  |
|  | Type | Numeric |  |  |
|  | Format | F40 |  |  |
|  | Measurement | Ordinal |  |  |
|  | Role | Input |  |  |
| Valid Values | 1 | A little of the time | 215 | 66.0% |
|  | 2 | Some of the time | 71 | 21.8% |
|  | 3 | Good part of the time | 21 | 6.4% |
|  | 4 | Most of the time | 19 | 5.8% |

| **Dprsn_9** | | | | |
| --- | --- | --- | --- | --- |
|  | | Value | Count | Percent |
| Standard Attributes | Position | 30 |  |  |
|  | Label | My heart beats faster than usual |  |  |
|  | Type | Numeric |  |  |
|  | Format | F40 |  |  |
|  | Measurement | Ordinal |  |  |
|  | Role | Input |  |  |
| Valid Values | 1 | A little of the time | 202 | 62.0% |
|  | 2 | Some of the time | 72 | 22.1% |
|  | 3 | Good part of the time | 37 | 11.3% |
|  | 4 | Most of the time | 15 | 4.6% |

| **Dprsn_10** | | | | |
| --- | --- | --- | --- | --- |
|  | | Value | Count | Percent |
| Standard Attributes | Position | 31 |  |  |
|  | Label | I get tired for no reason |  |  |
|  | Type | Numeric |  |  |
|  | Format | F40 |  |  |
|  | Measurement | Ordinal |  |  |
|  | Role | Input |  |  |
| Valid Values | 1 | A little of the time | 131 | 40.2% |
|  | 2 | Some of the time | 102 | 31.3% |
|  | 3 | Good part of the time | 48 | 14.7% |
|  | 4 | Most of the time | 45 | 13.8% |

| **Dprsn_11** | | | | |
| --- | --- | --- | --- | --- |
|  | | Value | Count | Percent |
| Standard Attributes | Position | 32 |  |  |
|  | Label | My mind is as clear as it used to be |  |  |
|  | Type | Numeric |  |  |
|  | Format | F40 |  |  |
|  | Measurement | Ordinal |  |  |
|  | Role | Input |  |  |
| Valid Values | 1 | Most of the time | 38 | 11.7% |
|  | 2 | Good part of the time | 53 | 16.3% |
|  | 3 | Some of the time | 103 | 31.6% |
|  | 4 | A little of the time | 132 | 40.5% |

| **Dprsn_12** | | | | |
| --- | --- | --- | --- | --- |
|  | | Value | Count | Percent |
| Standard Attributes | Position | 33 |  |  |
|  | Label | I find it easy to do the things I used to |  |  |
|  | Type | Numeric |  |  |
|  | Format | F40 |  |  |
|  | Measurement | Ordinal |  |  |
|  | Role | Input |  |  |
| Valid Values | 1 | Most of the time | 41 | 12.6% |
|  | 2 | Good part of the time | 48 | 14.7% |
|  | 3 | Some of the time | 120 | 36.8% |
|  | 4 | A little of the time | 117 | 35.9% |

| **Dprsn_13** | | | | |
| --- | --- | --- | --- | --- |
|  | | Value | Count | Percent |
| Standard Attributes | Position | 34 |  |  |
|  | Label | I am restless and cannot keep still |  |  |
|  | Type | Numeric |  |  |
|  | Format | F40 |  |  |
|  | Measurement | Ordinal |  |  |
|  | Role | Input |  |  |
| Valid Values | 1 | A little of the time | 160 | 49.1% |
|  | 2 | Some of the time | 102 | 31.3% |
|  | 3 | Good part of the time | 42 | 12.9% |
|  | 4 | Most of the time | 22 | 6.7% |

| **Dprsn_14** | | | | |
| --- | --- | --- | --- | --- |
|  | | Value | Count | Percent |
| Standard Attributes | Position | 35 |  |  |
|  | Label | I feel hopeful about the future |  |  |
|  | Type | Numeric |  |  |
|  | Format | F40 |  |  |
|  | Measurement | Ordinal |  |  |
|  | Role | Input |  |  |
| Valid Values | 1 | Most of the time | 74 | 22.7% |
|  | 2 | Good part of the time | 103 | 31.6% |
|  | 3 | Some of the time | 85 | 26.1% |
|  | 4 | A little of the time | 64 | 19.6% |

| **Dprsn_15** | | | | |
| --- | --- | --- | --- | --- |
|  | | Value | Count | Percent |
| Standard Attributes | Position | 36 |  |  |
|  | Label | I am more irritable than usual |  |  |
|  | Type | Numeric |  |  |
|  | Format | F40 |  |  |
|  | Measurement | Ordinal |  |  |
|  | Role | Input |  |  |
| Valid Values | 1 | A little of the time | 126 | 38.7% |
|  | 2 | Some of the time | 108 | 33.1% |
|  | 3 | Good part of the time | 58 | 17.8% |
|  | 4 | Most of the time | 34 | 10.4% |

| **Dprsn_16** | | | | |
| --- | --- | --- | --- | --- |
|  | | Value | Count | Percent |
| Standard Attributes | Position | 37 |  |  |
|  | Label | I find it easy to make decisions |  |  |
|  | Type | Numeric |  |  |
|  | Format | F40 |  |  |
|  | Measurement | Ordinal |  |  |
|  | Role | Input |  |  |
| Valid Values | 1 | Most of the time | 39 | 12.0% |
|  | 2 | Good part of the time | 78 | 23.9% |
|  | 3 | Some of the time | 113 | 34.7% |
|  | 4 | A little of the time | 96 | 29.4% |

| **Dprsn_17** | | | | |
| --- | --- | --- | --- | --- |
|  | | Value | Count | Percent |
| Standard Attributes | Position | 38 |  |  |
|  | Label | I feel that I am useful and needed |  |  |
|  | Type | Numeric |  |  |
|  | Format | F40 |  |  |
|  | Measurement | Ordinal |  |  |
|  | Role | Input |  |  |
| Valid Values | 1 | Most of the time | 100 | 30.7% |
|  | 2 | Good part of the time | 89 | 27.3% |
|  | 3 | Some of the time | 80 | 24.5% |
|  | 4 | A little of the time | 57 | 17.5% |

| **Dprsn_18** | | | | |
| --- | --- | --- | --- | --- |
|  | | Value | Count | Percent |
| Standard Attributes | Position | 39 |  |  |
|  | Label | My life is pretty full |  |  |
|  | Type | Numeric |  |  |
|  | Format | F40 |  |  |
|  | Measurement | Ordinal |  |  |
|  | Role | Input |  |  |
| Valid Values | 1 | Most of the time | 76 | 23.3% |
|  | 2 | Good part of the time | 102 | 31.3% |
|  | 3 | Some of the time | 91 | 27.9% |
|  | 4 | A little of the time | 57 | 17.5% |

| **Dprsn_19** | | | | |
| --- | --- | --- | --- | --- |
|  | | Value | Count | Percent |
| Standard Attributes | Position | 40 |  |  |
|  | Label | I feel that others would be better off if I were dead |  |  |
|  | Type | Numeric |  |  |
|  | Format | F40 |  |  |
|  | Measurement | Ordinal |  |  |
|  | Role | Input |  |  |
| Valid Values | 1 | A little of the time | 266 | 81.6% |
|  | 2 | Some of the time | 34 | 10.4% |
|  | 3 | Good part of the time | 17 | 5.2% |
|  | 4 | Most of the time | 9 | 2.8% |

| **Dprsn_20** | | | | |
| --- | --- | --- | --- | --- |
|  | | Value | Count | Percent |
| Standard Attributes | Position | 41 |  |  |
|  | Label | I still enjoy the things I used to do |  |  |
|  | Type | Numeric |  |  |
|  | Format | F40 |  |  |
|  | Measurement | Ordinal |  |  |
|  | Role | Input |  |  |
| Valid Values | 1 | Most of the time | 67 | 20.6% |
|  | 2 | Good part of the time | 80 | 24.5% |
|  | 3 | Some of the time | 109 | 33.4% |
|  | 4 | A little of the time | 70 | 21.5% |

| **Anxiety_cats** | | | | |
| --- | --- | --- | --- | --- |
|  | | Value | Count | Percent |
| Standard Attributes | Position | 44 |  |  |
|  | Label | Anxiety symptom severity |  |  |
|  | Type | Numeric |  |  |
|  | Format | F8 |  |  |
|  | Measurement | Ordinal |  |  |
|  | Role | Input |  |  |
| Valid Values | 0 | Normal | 89 | 27.3% |
|  | 1 | Mild | 144 | 44.2% |
|  | 2 | Moderate | 53 | 16.3% |
|  | 3 | Severe | 40 | 12.3% |

| **Dprsn_cats** | | | | |
| --- | --- | --- | --- | --- |
|  | | Value | Count | Percent |
| Standard Attributes | Position | 45 |  |  |
|  | Label | Depression symptom severity |  |  |
|  | Type | Numeric |  |  |
|  | Format | F8 |  |  |
|  | Measurement | Ordinal |  |  |
|  | Role | Input |  |  |
| Valid Values | 0 | Normal | 246 | 75.5% |
|  | 1 | Mild | 71 | 21.8% |
|  | 2 | Moderate | 7 | 2.1% |
|  | 3 | Severe | 2 | 0.6% |
